# Executive Functioning and Mental Health in Adolescent Kidney Transplant Candidates During the COVID‐19 Pandemic

**DOI:** 10.1111/petr.70152

**Published:** 2025-08-01

**Authors:** Finola E. Kane‐Grade, Danielle Glad, Christopher Anzalone, Michael D. Evans, Sarah Kizilbash, Lidan Gu

**Affiliations:** ^1^ Division of Clinical Behavioral Neuroscience, Department of Pediatrics University of Minnesota Medical School Minneapolis Minnesota USA; ^2^ Department of Neurology Medical College of Wisconsin Milwaukee Wisconsin USA; ^3^ Department of Psychiatry and Behavioral Sciences Boston Children's Hospital, Harvard Medical School Boston Massachusetts USA; ^4^ Clinical and Translational Science Institute, University of Minnesota Minneapolis Minnesota USA; ^5^ Division of Pediatric Nephrology, Department of Pediatrics University of Minnesota Medical School Minneapolis Minnesota USA

**Keywords:** adolescents, COVID‐19 pandemic, executive functioning, kidney failure, mental health

## Abstract

**Background:**

Pediatric patients with kidney failure are at increased risk for executive functioning and mental health concerns. The COVID‐19 pandemic induced prolonged global stress, potentially intensifying these concerns for patients. This study aimed to determine if pediatric kidney transplant candidates evaluated during the pandemic exhibited greater executive functioning and mental health concerns compared to candidates evaluated before the pandemic.

**Method:**

We retrospectively evaluated 43 pediatric kidney transplant candidates (ages 3–17) who completed pretransplant neuropsychological evaluations between 2017 and 2022. The cohort was divided into two groups by evaluation era (before pandemic [*n* = 21]; during pandemic [*n* = 22]). Executive function and mental health were compared across groups using analysis of variance adjusting for covariates. Analyses were stratified by age at evaluation (adolescents: 13–17; preadolescents: 3–12 years), and age‐by‐era interaction was assessed.

**Results:**

Our cohort included 23 adolescents (*M*_*age*_ = 15.4 years, SD = 1.2) and 20 preadolescents (*M*_*age*_ = 8.8 years, SD = 3.3); 56% of candidates were female and 67% were white. The associations between era and emotional regulation, cognitive regulation, internalizing, and externalizing mean scores were significantly modified by age (interaction *p*‐values < 0.05). Adolescents, but not preadolescent candidates, evaluated during the pandemic showed significantly greater difficulties with emotional regulation (adjusted (adj) mean difference: −14.3; *p* = 0.01), cognitive regulation (adj difference: −15.7; *p* = 0.003), internalizing (adj difference: −15.8; *p* = 0.0008); and externalizing concerns (adj difference: −9.6; *p* = 0.009) versus adolescents evaluated before the pandemic.

**Conclusion:**

Adolescent candidates evaluated during the COVID‐19 pandemic had significantly higher executive functioning and mental health concerns compared to those evaluated before the pandemic; however, no significant differences were found in the mean scores for preadolescent candidates.

## Graphical abstract

Adolescent kidney transplant candidates, not preadolescent candidates, exhibited greater executive functioning difficulties and mental health concerns during the COVID‐19 pandemic compared to adolescent candidates evaluated before the pandemic. Clinicians and providers should monitor for the potential impact of life stressors and recommend opportunities to promote resilience through targeted interventions.

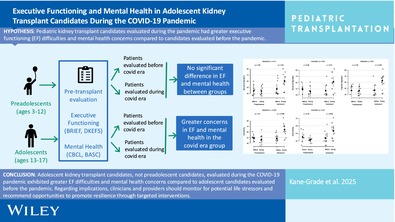

**Abbreviations**
BASC: Behavioral Assessment Scale for Children
BRIEF: Behavior Rating Inventory of Executive Function
CAKUT: Congenital Anomalies of the Kidney and Urinary Tract
CBCL: Child Behavior Checklist
CKD: Chronic Kidney Disease
D‐KEFS: The Delis‐Kaplan Executive Functioning System
EF: Executive Functioning

## 1 Introduction

Over recent decades, medical advancements have significantly improved the long‐term survival of children and adolescents with kidney failure. With enhanced survival, the focus has increasingly shifted toward optimizing growth and neurocognitive development. Neurocognitive deficits and mental health challenges are well documented in children with chronic kidney disease (CKD). Children with CKD tend to score lower on standardized tests of intelligence and academic achievement [1, 2]. Additionally, children with CKD are more likely to experience executive functioning (EF) impairments, such as reduced attention regulation and inhibitory control [3]. Mental health disorders, such as depression and anxiety, are also common in this population [4].

Pediatric kidney transplant candidates are at increased risk of experiencing EF deficits due to risk factors related to their health status and factors such as limited interactions with their peers, school, and community. As cognitive impacts and reduced enrichment opportunities may begin with CKD onset, EF deficits likely begin to develop before transplant [3]. Prior research indicates that youth with EF impairments have more barriers to adherence, lower rates of medication adherence, and more academic challenges [5, 6]. Further, children with CKD experience a range of disease‐associated challenges that generate complex psychosocial effects and increased stressors (e.g., medical interventions), which can lead to lower quality of life and mental health challenges. The prevalence of depression and anxiety is common in this population, with prevalence rates ranging from 17%–36% and 26%–36%, respectively [4]. Importantly, negative health outcomes (e.g., increased readmission rates) and increased financial and emotional costs can occur when mental illness is not identified and treated in this population [7].

As a significant life stressor, the COVID‐19 pandemic may have exacerbated the EF and mental health concerns of already vulnerable pediatric kidney transplant candidates by inducing prolonged stress, isolation, and disruptions to day‐to‐day routine. The combined medical‐related stressors and life stressors (e.g., stressors related to the COVID‐19 pandemic) may have presented unique challenges for this population. Although the COVID‐19 pandemic has been shown to exacerbate the vulnerability of children with various other chronic medical conditions to psychopathology [8, 9], no study has evaluated the effects of the COVID‐19 pandemic on the mental health and EF of children and adolescents with kidney failure.

Adolescence is a time of significant brain development (e.g., structural alterations in the central nervous system and cortico‐limbic regions), cognitive maturation, and social–emotional growth. Solid organ transplant recipients experience a period of unique vulnerability during adolescence, when normative developmental changes intersect with health‐related variables to influence psychological health. Existing research suggests that social isolation and loneliness are associated with increased anxiety and depression among adolescents [10]. Therefore, adolescent kidney transplant candidates may be at particularly heightened risk for EF and mental health difficulties, especially during a pandemic.

This study aimed to investigate whether pediatric kidney transplant candidates evaluated during the COVID‐19 pandemic exhibited different mental health and EF concerns compared to candidates evaluated before the pandemic. In addition, specific behavior variables that were associated with mental health concerns and EF in this population were examined to determine which mental health component affects daily EF most. Based on the greater susceptibility of children in general to psychopathology due to COVID‐19 [11], we hypothesized that (1) pediatric kidney transplant candidates evaluated during the COVID‐19 pandemic would have greater mental health concerns and lower EF, on average, compared to candidates evaluated prior to the COVID‐19 pandemic; and (2) adolescent candidates would be at a higher risk for mental health and EF difficulties compared to preadolescent candidates due to the greater susceptibility of adolescent candidates to the adverse consequences of social isolation. This is an important study as it characterizes the EF and mental health profile of pediatric kidney transplant candidates in the setting of a prolonged life stressor, highlighting their vulnerability and the need for support strategies.

## 2 Methods

### 2.1 Study Design

This was a single‐center, retrospective, observational study. This project was approved by the Institutional Review Board at the University of Minnesota‐Twin Cities (IRB #00017782).

### 2.2 Study Population

At our institution, all kidney transplant candidates are routinely referred for neuropsychological testing as part of our institutional pretransplant evaluation protocol in accordance with KDIGO recommendations [12] for young children and as a result of a strong collaboration between the transplant team and the neuropsychology team. In this study, we identified all pediatric kidney transplant candidates (ages 3–17 years) who underwent pretransplant neuropsychological evaluations per our institutional protocol between 2017 and 2022. Patients who opted out of research were not included. Further, patients who were younger than 3 years at the time of neuropsychological evaluation were excluded from this study given that toddlers in our clinic typically would not complete objective or parent‐reported EF measures. We then divided the study period into before the COVID‐19 pandemic (2017–2019) and during the COVID‐19 pandemic (2020–2022) eras. This yielded a cohort of 21 patients evaluated before the pandemic (9 preadolescents ages 3–12 years; 12 adolescents ages 13–17 years) and 22 patients evaluated during the pandemic (11 preadolescents ages 3–12; 11 adolescents ages 13–17 years). As part of the evaluation, parents or caregivers were asked to complete questionnaires related to mental health and EF. Some patients also completed direct, performance‐based measures assessing EF when it was included in their test battery and was age‐appropriate.

### 2.3 Outcome Measures

#### 2.3.1 Executive Functioning

Caregivers completed either the Behavior Rating Inventory of Executive Function—Preschool Version (BRIEF‐P [13]) for children ages 3–5 years or the Behavior Rating Inventory of Executive Function—2nd Edition (BRIEF‐2 [14]) for children 6 years or older. The BRIEF‐P and BRIEF‐2 are 63‐item and 86‐item standardized caregiver rating scales of behavioral manifestations of EF, commonly thought of as day‐to‐day executive skills. Items are rated on a 3‐point scale including “Never,” “Sometimes,” and “Often.” The Global Executive Composite (GEC) from the BRIEF‐P and BRIEF‐2 was used to determine the overall caregiver‐reported EF. We used an additional three composite scales from the measures, including Behavior Regulation (BR), Emotion Regulation (ER), and Cognitive Regulation (CR). The scores are calculated based on caregiver responses and are presented as age‐normed *T*‐scores (*M* = 50, SD = 10). Higher scores indicate a greater quantity and frequency of behaviors commonly associated with weaknesses in executive function. A *T*‐score < 60 indicates nonclinical symptoms, a *T*‐score between 60 and 64 indicates a mild elevation, a *T*‐score between 65 and 69 indicates a potential clinical elevation, and a *T*‐score > 70 indicates a clinical elevation (i.e., clinical concern for EF skill deficits). The BRIEF‐P and BRIEF‐2 scores have shown good test–retest reliability, internal consistency, and validity with other similar rating scale measures [15]. The parents of 16 preadolescent candidates (80%) and 20 adolescents (87%) completed the BRIEF measure.

The Delis‐Kaplan Executive Functioning System (D‐KEFS [16]) Trail Making Test is a performance‐based test for EF for ages 8 years and above. The Number‐Letter Sequencing condition was used as an objective measure of EF skills. Number‐Letter Sequencing evaluated EF skills such as cognitive flexibility, working memory, and processing speed. Performance is provided as a scaled score (*M* = 10, SD = 3). The D‐KEFS tests tend to display moderately good internal consistency coefficients and good test–retest reliability, and the validity of the Trail Making Test has been demonstrated in numerous neuropsychological studies conducted during the past 50 years or more [17]. Ten preadolescent candidates (50%) and 20 adolescents (87%) completed the D‐KEFS.

#### 2.3.2 Mood and Behavior

Internalizing and externalizing symptomatology was assessed using scales from the Child Behavior Checklist (CBCL [18]) and Behavioral Assessment Scale for Children—3rd Edition (BASC‐3 [19]), both of which are caregiver report behavioral and emotional functioning questionnaires. The questionnaire was selected based on provider preference at time of neuropsychological evaluation. The CBCL is among the most well‐established, empirically supported questionnaires to assess child psychopathology symptoms [20]. Symptoms are rated on a 3‐point scale of “Not True,” “Sometimes True,” and “Very True or Often True.” *T*‐scores (*M* = 50, SD = 10) are provided for composite scores. The BASC‐3 composites and subscales are also well established. *T*‐scores (*M* = 50, SD = 10) are provided for composite scores and domain‐level scores. For the Clinical Scales, scores ranging from 60 to 69 are considered to be in the “at‐risk” range (i.e., subclinical elevation) and scores of 70 or higher are considered “clinically significant.” Specific subscales that represent internalizing concerns and externalizing concerns were included in further analyses. These subscales include aggression, attention, anxiety, and depression scales on both the CBCL and BASC‐3. Additionally, because we were interested in potential impact from social isolation during the pandemic, subscales that represent social functioning were included and combined as one variable (the BASC‐3 Social Skills *T*‐scores were reversed to show consistent direction with the CBCL Social Problems *T*‐scores). Seventeen preadolescents (85%) and 22 adolescents (96%) had caregiver report CBCL or BASC.

### 2.4 Statistical Analyses

Descriptive statistics of participant characteristics were summarized using frequencies and percentages or means and standard deviations. We divided the study cohort into two groups by the era of evaluation (prepandemic era [2017–2019] and COVID‐19 pandemic era [2020–2022]). Age at evaluation was categorized into preadolescent (ages 3–12 years) and adolescent (ages 13–17 years) categories. Executive function and mental health (internalizing concerns, externalizing concerns, behavioral regulation, emotion regulation, and cognitive regulation) were compared across groups using analysis of variance (ANOVA). As noted in the introduction section, previous literature indicated that medical and life stressors impact mental health and executive functioning. We wanted to control for potential associations between demographic/medical characteristics and executive functioning/mental health outcomes. Thus, these models included covariates adjusting for age at evaluation, era, age‐by‐era interaction term, sex, race, kidney failure type, dialysis type, and insurance type. To identify the most informative predictors of caregiver‐reported EF [BRIEF Global Executive Composite score], specific subscales such as social problems, aggression, attention, anxiety, and depression were included in variable selection using LASSO regression with leave‐one‐out cross‐validation. Results are reported using means with 95% confidence intervals. Analyses were performed using R version 4.2.2 including the packages glmnet version 4.1–4 [21] and emmeans version 1.8.2 [22].

## 3 Results

### 3.1 Description of Participants

Table 1 provides a description of participant characteristics. Among the preadolescent group (*n* = 20), the mean age for developing kidney failure was 8.28 years (SD = 3.43), and the mean age for completing neuropsychological evaluation was 8.75 years (SD = 3.34). Among the adolescent group (*n* = 23), the mean age for developing kidney failure was 15.40 years (SD = 1.2 years), and the mean age for completing neuropsychological evaluation was 15.43 years (SD = 1.24).

**TABLE 1:** Demographics characteristics.

Variable	Level	Before pandemic	During pandemic	Total
*n*		21	22	43
Age group (%)	Preadolescent	9 (42.9)	11 (50.0)	20 (46.5)
	Adolescent	12 (57.1)	11 (50.0)	23 (53.5)
Age (mean [SD])	Preadolescent	8.78 (3.73)	8.73 (3.17)	8.75 (3.34)
	Adolescent	15.50 (1.17)	15.36 (1.36)	15.43 (1.24)
Sex (%)	F	10 (47.6)	14 (63.6)	24 (55.8)
	M	11 (52.4)	8 (36.4)	19 (44.2)
Race (%)	American Indian or Alaskan Native	2 (9.5)	5 (22.7)	7 (16.3)
	Asian or Asian American	3 (14.3)	2 (9.1)	5 (11.6)
	Black or African American	1 (4.8)	1 (4.5)	2 (4.7)
	White	15 (71.4)	14 (63.6)	29 (67.4)
Insurance type (%)	Private	15 (71.4)	8 (36.4)	23 (53.5)
	Public	6 (28.6)	14 (63.6)	20 (46.5)
Disease category (%)	CAKUT	9 (42.9)	6 (27.3)	15 (34.9)
	Nephrotic syndrome	6 (28.6)	4 (18.2)	10 (23.3)
	Other	6 (28.6)	12 (54.5)	18 (41.9)
Dialysis category (%)	None	8 (38.1)	6 (27.3)	14 (32.6)
	Hemodialysis	10 (47.6)	10 (45.5)	20 (46.5)
	Peritoneal	3 (14.3)	5 (22.7)	8 (18.6)
	Both	0 (0.0)	1 (4.5)	1 (2.3)
Dialysis length by months (mean [SD])		8.44 (9.18)	8.09 (5.47)	8.36 (7.34)

The demographic characteristics of the before pandemic and during pandemic groups are also displayed in Table 1. Specifically, the mean age of preadolescents was 8.78 years (SD = 3.73) before the pandemic and 8.73 years (SD = 3.17) during the pandemic. The mean age of adolescents was 15.50 years (SD = 1.17) before the pandemic and 15.36 years (SD = 1.36) during the pandemic. Ten participants (47.6%) from the before pandemic group and 14 participants (63.6%) from the pandemic group were female. Fifteen (71.4%) from the before pandemic group and 14 (63.6%) from the pandemic group were White. The most common underlying cause of kidney disease in the before pandemic group (42.9%) was congenital anomalies of the kidney and urinary tract (CAKUT), while the most common underlying disease in the pandemic group (54.5%) belongs to the “Other” category which includes nephritis, hemolytic uremic syndrome, cortical necrosis, cystinosis, Wilm's tumor, cystic kidney disease, nephropathy, oxalate, Wegener's granulomatosis, and kidney failure due to unknown causes. The majority of participants from the before pandemic group (61.9%) and the during pandemic group (72.7%) were receiving dialysis at the time of their neuropsychological evaluation. The mean length of dialysis was 8.44 months (SD = 9.18) in the before group and 8.09 months (SD = 5.47) in the during pandemic group.

### 3.2 Mental Health and Executive Functioning Outcomes

Parent‐reported overall BRIEF‐2 score ranged between 36 and 80 for behavior regulation, 38–82 for emotion regulation, and 38–82 for cognitive regulation. The BASC and CBCL score ranges included an internalizing index of 36–86 and an externalizing index of 34–71. On average, patients evaluated before the pandemic and during the pandemic were both reported to have mental health and EF in the normal range. When comparing outcome measures between patients evaluated before and those evaluated during the COVID‐19 pandemic, no significant differences were found in caregiver reports across groups for internalizing concerns (before vs. during mean scores = 49.78 vs. 55.10; *p* = 0.14), externalizing concerns (before vs. during mean scores = 45.06 vs. 47.24, *p* = 0.45), behavior regulation (before vs. during mean scores = 48.60 vs. 53.47; *p* = 0.25), emotion regulation (before vs. during mean scores = 46.94 vs. 51.95; *p* = 0.23), or cognitive regulation (before vs. during mean scores = 48.88 vs. 52.95; *p* = 0.33).

#### 3.2.1 Executive Functioning and Mental Health by Age at Evaluation

**FIGURE 1:**
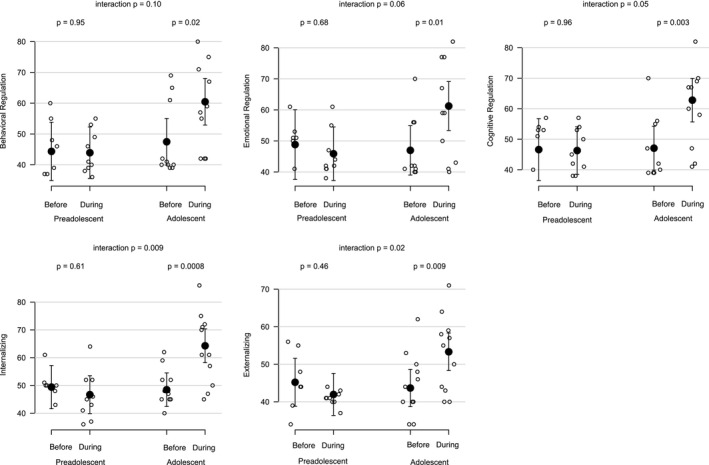
Adolescent pretransplant candidates showed greater executive functioning difficulties and mental health concerns during the COVID‐19 pandemic, in comparison with their before pandemic cohort. However, there were no significant difference in these domains between the before and during pandemic groups in the preadolescent candidates.

When outcome measures were compared within the preadolescent group (as shown in Table 2), parents reported their children's mental health and EF scores in the “normal” range (i.e., mean scores between 40 and 59) both before and during the pandemic. Regarding the adolescent group (aged 13–17), the before pandemic cohort was reported to have mental health and EF scores in the normal range. However, adolescents during the pandemic had mildly elevated difficulties with emotional regulation (*M* = 59.5), cognitive regulation (*M* = 60.3) scores, and global EF (*M* = 60.5), as well as “at‐risk” internalizing symptomatology (*M* = 63.2). Further, adolescents during the pandemic had behavioral regulation (*M* = 59) and externalizing symptomatology (*M* = 52.8) scores that were in the normal range. Figure 1 displays EF and mental health scores for youth evaluated before and during the pandemic, stratified by age at evaluation.

**TABLE 2:** Executive functioning and mental health based on age and pandemic era.

Age	Era	Unadjusted				Adjusted for gender, race, ESKD cause, dialysis type, insurance			
		Mean (95% CI)	Before—During		Interaction *p*‐value (age‐by‐covid)	Mean (95% CI)	Before—During		Interaction *p*‐value (age‐by‐covid)
			Mean (95% CI)	*p*			Mean (95% CI)	*p*	
**Behavioral regulation**									
Preadolescent	Before	46.0 (37.4, 54.6)	1.9 (−9.6, 13.4)	0.74	0.09	44.3 (34.9, 53.8)	0.4 (−12.6, 13.5)	0.95	0.10
Preadolescent	During	44.1 (36.5, 51.7)				43.9 (35.5, 52.3)			
Adolescent	Before	47.6 (40.4, 54.8)	−11.4 (−21.6, −1.2)	0.03		47.5 (39.9, 55.0)	−13.0 (−23.7, −2.3)	0.02	
Adolescent	During	59.0 (51.8, 66.2)				60.5 (52.9, 68.0)			
**Emotional regulation**									
Preadolescent	Before	51.2 (41.9, 60.4)	5.5 (−6.4, 17.4)	0.36	0.03	48.8 (37.6, 60.1)	3.0 (−11.5, 17.4)	0.68	0.06
Preadolescent	During	45.7 (38.1, 53.2)				45.9 (37.2, 54.5)			
Adolescent	Before	47.5 (40.3, 54.7)	−12.0 (−22.1, −1.9)	0.02		47.0 (39.0, 54.9)	−14.3 (−25.3, −3.2)	0.01	
Adolescent	During	59.5 (52.3, 66.7)				61.2 (53.4, 69.1)			
**Cognitive regulation**									
Preadolescent	Before	51.3 (42.9, 59.8)	4.9 (−6.1, 15.8)	0.37	0.01	46.6 (36.5, 56.7)	0.3 (−12.7, 13.4)	0.96	0.05
Preadolescent	During	46.4 (39.5, 53.4)				46.3 (38.5, 54.1)			
Adolescent	Before	46.6 (40.0, 53.2)	−13.7 (−23.0, −4.4)	0.005		47.1 (39.9, 54.3)	−15.7 (−25.7, −5.8)	0.003	
Adolescent	During	60.3 (53.7, 66.9)				62.8 (55.7, 69.9)			
**Internalizing**									
Preadolescent	Before	50.4 (43.5, 57.4)	4.2 (−4.8, 13.3)	0.35	0.004	49.4 (41.6, 57.2)	2.7 (−8.1, 13.5)	0.61	0.009
Preadolescent	During	46.2 (40.4, 52.0)				46.7 (39.9, 53.5)			
Adolescent	Before	49.4 (43.8, 54.9)	−13.8 (−21.6, −6.0)	0.001		48.5 (42.4, 54.5)	−15.8 (−24.4, −7.2)	0.0008	
Adolescent	During	63.2 (57.6, 68.7)				64.3 (58.2, 70.4)			
**Externalizing**									
Preadolescent	Before	45.7 (39.7, 51.8)	4.6 (−3.3, 12.5)	0.24	0.02	45.2 (38.8, 51.6)	3.2 (−5.6, 12.1)	0.46	0.02
Preadolescent	During	41.1 (36.0, 46.2)				42.0 (36.4, 47.6)			
Adolescent	Before	44.6 (39.8, 49.5)	−8.2 (−15.0, −1.4)	0.02		43.7 (38.7, 48.6)	−9.6 (−16.7, −2.6)	0.009	
Adolescent	During	52.8 (48.0, 57.6)				53.3 (48.3, 58.3)			
**D KEFS trail making number letter switching**									
Preadolescent	Before	8.0 (4.2, 11.8)	−0.3 (−5.5, 4.8)	0.90	0.64	11.1 (6.1, 16.0)	2.7 (−4.0, 9.3)	0.41	0.24
Preadolescent	During	8.3 (4.9, 11.8)				8.4 (4.2, 12.6)			
Adolescent	Before	6.6 (3.9, 9.3)	−1.8 (−5.8, 2.1)	0.34		5.8 (2.9, 8.8)	−1.7 (−5.7, 2.3)	0.37	
Adolescent	During	8.4 (5.6, 11.3)				7.6 (4.6, 10.5)			

#### 3.2.2 Behavior Regulation

Adolescents evaluated during the COVID‐19 pandemic showed greater caregiver‐reported behavior regulation difficulties compared to adolescents evaluated before the COVID pandemic (adj mean difference = −13.0; *p* = 0.02). As shown in Table 2, we observed no significant difference in the mean scores of preadolescent candidates for cognitive regulation symptoms (adj mean difference: 0.4; *p* = 0.95). However, the age at evaluation‐by‐era interaction for behavior regulation did not achieve statistical significance (adj *p* = 0.1).

#### 3.2.3 Emotional Regulation

Adolescents evaluated during the COVID‐19 pandemic showed greater caregiver‐reported emotional regulation difficulties compared to adolescents evaluated before the COVID pandemic (adj mean difference = −14.3; *p* = 0.01). We observed no significant difference in the mean scores of preadolescent candidates for cognitive regulation symptoms (adj mean difference: 3.0; *p* = 0.68). The age at evaluation‐by‐era interaction for emotional regulation trended toward statistical significance (adj *p* = 0.06).

#### 3.2.4 Cognitive Regulation

We observed a significant interaction between age at evaluation and evaluation era for cognitive regulation symptoms (age‐by‐era interaction *p*‐value (adj) = 0.05). Adolescents evaluated during the COVID‐19 pandemic showed greater caregiver‐reported cognitive regulation difficulties compared to adolescents evaluated before the COVID pandemic (adj mean difference = −15.7; *p* = 0.003). However, we observed no significant difference in the mean scores of preadolescent candidates for cognitive regulation symptoms (adj mean difference: 0.3; *p* = 0.96).

#### 3.2.5 Internalizing Symptoms

We observed a significant interaction between age at evaluation and evaluation era for internalizing symptoms (age‐by‐era interaction *p*‐value (adj) = 0.009). Adolescents evaluated during the COVID‐19 pandemic showed greater caregiver‐reported difficulties compared to adolescents evaluated before the COVID pandemic on internalizing symptoms (adjusted (adj) mean difference = −15.8; *p* < 0.001). However, we observed no significant difference in the mean scores of preadolescent candidates for internalizing symptoms (adj mean difference: 2.7; *p* = 0.61).

#### 3.2.6 Externalizing Symptoms

We observed a significant interaction between age at evaluation and evaluation era for externalizing symptoms (age‐by‐era interaction *p*‐value (adj) = 0.02). Adolescents evaluated during the COVID‐19 pandemic showed greater caregiver‐reported difficulties compared to adolescents evaluated before the COVID pandemic on externalizing symptoms (adj mean difference = −9.6; *p* = 0.009). However, we observed no significant difference in the mean scores of preadolescent candidates for externalizing symptoms (adj mean difference: 3.2; *p* = 0.46).

#### 3.2.7 Direct EF Performance (DKEFS)

We observed no significant difference in D KEFS results between individuals evaluated before and those evaluated during the pandemic, regardless of the age at evaluation (*ps* > 0.05).

### 3.3 Prediction of Daily Executive Functioning Concerns

**FIGURE 2:**
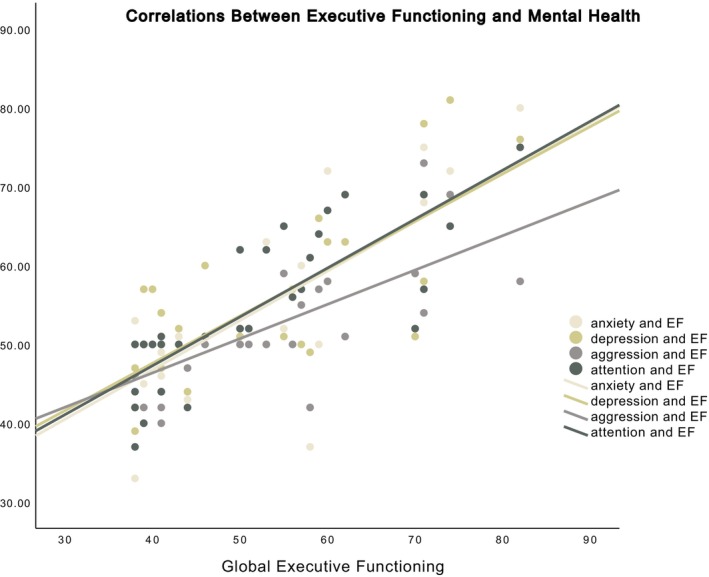
Correlations between caregiver‐reported mental health concerns (depression, anxiety, aggression, and attention) and the global EF index were all higher than 0.7.

Further examination of the mental health and EF concerns reported by caregivers showed high correlations between mental health concerns and reported EF difficulties. Correlations between caregiver‐reported mental health concerns (depression, anxiety, aggression, and attention) and the caregiver‐reported global EF index (*n* = 31) were all higher than 0.7 (*p*s < 0.001), as shown in Figure 2. However, the correlation between social problems and the global EF index was moderate at 0.55. LASSO regression identified inattention (coefficient = 0.54), anxiety (coefficient = 0.16), and aggression (coefficient = 0.08) as predictors of the global EF index, while depression, age, and social problems were not selected by the model.

## 4 Discussion

The primary objective of this study was to compare mental health and EF of pediatric kidney transplant recipients evaluated during the COVID‐19 pandemic with those evaluated before the pandemic. Contrary to our first hypothesis, pediatric kidney transplant candidates evaluated during the pandemic overall did not show greater mental health and EF concerns than candidates evaluated before the pandemic. However, we confirmed the second hypothesis. Adolescents kidney transplant candidates, not the preadolescent candidates, were reported by caregivers to have greater EF and mental health concerns during the COVID‐19 pandemic compared to adolescents evaluated prior to the pandemic. On average, the reported concerns of global EF, emotion regulation, cognitive regulation, and internalizing concerns were moderately elevated (in the “at‐risk” range) in the adolescent group during the COVID‐19 pandemic, which confirmed what we predicted from the literature that both mental health and EF can be impacted by a life stressor like the COVID‐19 pandemic [23, 24]. These study findings highlight how the combined medical‐related stressors and life stressors (e.g., stressors related to the COVID‐19 pandemic) can exacerbate mental health and EF challenges, particularly during the developmental stage of adolescence.

The study findings showed that the impact of multiple/accumulated stressors on transplant candidates may not be limited to mental health. Executive functioning can be impacted, which can also affect day‐to‐day functioning and medical care. Although life stressors are inevitable, chronic stress exposure, such as continuous stress triggered by a global pandemic or a chronic medical condition, can become “toxic” and lead to negative mental health and physical health outcomes [25]. Moreover, it can also induce structural and functional changes in the developing brain [26]. Repeated stress exposures are a risk factor for the development of psychopathology and have considerable impacts on the amygdala and frontal cortex that are linked to mental health and EF difficulties [26, 27]. The negative psychological impacts of the COVID‐19 pandemic on adolescents can become a “double hit” when combined with medical stress associated with advanced chronic kidney disease. Our results underscore the importance of considering, monitoring, and treating potential consequences of chronic stress exposure.

Additionally, the study findings demonstrated different stress responses in different age groups. Adolescence is a vulnerable developmental window, with structural alterations happening in the central nervous system (CNS) and cortico‐limbic regions, and any stressful challenges during this critical period can trigger short‐and long‐term physiological, cognitive, and behavioral disruption. Importantly, adolescence is also a period of considerable plasticity [28]. Thus, treatment considerations for adolescent kidney transplant candidates and recipients should take into account their susceptibility to EF difficulties specifically in the setting of prolonged stressors, as EF skills are critical for navigating the complex posttransplant journey [29, 30]. Hence, it is important to assess the effects of other life stressors in addition to medical‐related stressors on the daily functioning of pediatric kidney transplant candidates [31]. This study highlights the cumulative effect of developmental stage (i.e., adolescence), a chronic medical condition (i.e., kidney failure), and a significant life stressor (e.g., a global pandemic) on EF, mood, and behavior.

Our study indicated that adolescents with kidney failure struggled more with EF abilities during the COVID‐19 pandemic compared to adolescents evaluated prior to the pandemic. However, these differences were only observed based on the caregiver reports of EF but not based on adolescents' direct performance on EF. This discrepancy between objective measures and caregiver report of EF has been documented previously in the literature [32, 33]. Caregivers provide the opportunity to elucidate a young individual's daily behavior within authentic contexts, and therefore the input of parents is necessary to understand EF abilities in real‐life contexts [34].

Further, our results indicated that attention difficulties and anxiety concerns predicted increased EF difficulties. Adolescent candidates' daily EF difficulties observed in this study may have been associated with fears and uncertainties (e.g., related to the COVID‐19 pandemic and the kidney transplantation process) demanding their cognitive attention. This finding provided suggestions for potential interventions to address EF difficulties in the context of life stressors. To address the fear and anxiety, adolescents transplant candidates will likely benefit from receiving direct and clear communication from healthcare providers regarding their health status and treatments to increase predictability and decrease uncertainty. Further, fear and vigilance can be addressed by helping adolescents recognize any level of control (e.g., medication adherence) they might have over their health outcomes.

Given that EF is linked to medication adherence and quality of life [35], adolescent kidney transplant candidates who have to manage additional life stressors may be more vulnerable to medication nonadherence after kidney transplantation, requiring additional monitoring and support. Approximately 30% of pediatric kidney transplant recipients have difficulty adhering to their medication regimen posttransplantation [36], with nonadherence rates being threefold higher in adolescents than in younger preadolescents. Further, opportunities for promoting resilience through interventions that target internalizing symptoms and EF are likely to be beneficial to this population [37], such as individual or group cognitive behavioral therapy (CBT). Furthermore, having a strong social support system, including a responsive caregiving environment, can also serve as a protective factor for mitigating the negative impact of repeated stress exposures [38]. Referrals to mental health providers, psychiatric medication management, and monitoring the impact of mental health difficulties on medical care should be considered as appropriate. A developing body of research has highlighted the importance of regular assessment of psychological health in children with chronic illness as well as their families. Providers need to be aware that this cohort may be more vulnerable, even as they age into adulthood, from mental health and EF standpoints. Future research should aim to examine how strategies such as close monitoring and additional support can reduce the potential risk of significant mental health difficulties or nonadherence.

While the current study is the first of its kind, several limitations of this work warrant consideration. First, our sample size is small; however, it is consistent with previous research in children with kidney failure [39, 40]. Second, this is a single‐center study, limiting the generalizability of the results. Further, our study does not include measures of stress or direct effects of COVID‐19. Additionally, our data is cross‐sectional. Given that our study is not longitudinal, we are not assessing youths' changes in executive functioning or mental health throughout the transplant process. While this is a direction for future research, our work has important implications. Previous research has found that youth with (parent‐reported) executive dysfunction and mental health challenges experience lower quality of life [41, 42]. As medical advances have led to improved patient and graft survival, quality of life has been highlighted for its important contribution to and as a component of psychological functioning, which in turn impacts medical outcomes. Results from this investigation highlight the vulnerability of this patient population to EF and mental health difficulties, informing the need for regular monitoring and support to improve quality of life and medical outcomes for this population.

In summary, adolescents with kidney failure evaluated during the COVID‐19 pandemic exhibited greater EF and mental health concerns compared to adolescents evaluated before the pandemic. Significant clinical implications follow the results of this study. Understanding how adolescent patients with kidney failure responded to the COVID‐19 stressor is important as it allows for a comprehensive understanding of the potential repercussions not only on their mental well‐being but also on their daily functionality and self‐regulatory capacities. When possible, clinicians and providers should monitor for potential life stressors and recommend opportunities to promote resilience to medical‐related stress through targeted interventions (e.g., focused on threat perception, emphasis on a sense of control, and a sensitive social support system).

## Disclosure

Data analyses were performed by Michael Evans, who is supported by the National Institutes of Health's National Center for Advancing Translational Sciences, grant UL1TR002494. Manuscript preparation was supported by Finola Kane‐Grade's National Science Foundation Graduate Research Fellowship.

## Conflicts of Interest

The authors declare no conflicts of interest.

## Data Availability

Research data are not shared.
